# Genome-Wide RNAi Longevity Screens in *Caenorhabditis elegans*

**DOI:** 10.2174/138920212803251391

**Published:** 2012-11

**Authors:** Melana E Yanos, Christopher F Bennett, Matt Kaeberlein

**Affiliations:** 1 Department of Pathology, University of Washington, Seattle, WA, USA; 2 Department of Psychology, University of Washington, Seattle, WA, USA; 3 Molecular and Cellular Biology Program, University of Washington, Seattle, WA, USA; 4 Institute of Aging Research, Guangdong Medical College, Dongguan 523808, China

**Keywords:** Dietary restriction, FOXO, Genomic, Longevity, IGF-1, Insulin, Mitochondria.

## Abstract

Progress in aging research has identified genetic and environmental factors that regulate longevity across species. The nematode worm *Caenorhabditis* *elegans* is a genetically tractable model system that has been widely used to investigate the molecular mechanisms of aging, and the development of RNA interference (RNAi) technology has provided a powerful tool for performing large-scale genetic screens in this organism. Genome-wide screens have identified hundreds of genes that influence lifespan, many of which fall into distinct functional classes and pathways. The purpose of this review is to summarize the results of large-scale RNAi longevity screens in *C. elegans*, and to provide an in-depth comparison and analysis of their methodology and most significant findings.

## INTRODUCTION

### A Brief History of Longevity Genetics Research in *C. elegans*

Progress in aging research has identified genetic and environmental factors that regulate longevity across species [1-3]. The nematode worm *Caenorhabditis elegans* has become an invaluable model system for investigating the molecular mechanisms of aging and longevity, offering the advantages of its relatively low cost, short lifespan, and conservation of key nutrient and stress-responsive signaling pathways in mammals. 

Prior to assuming a significant role in aging research, however, *C. elegans* had already been established as a useful model organism for studies in genetics and molecular biology. The development of a modern laboratory approach utilizing *C. elegans* can largely be attributed to the pioneering research of Sydney Brenner and John Sulston, whose work on the identification of *C. elegans* genes and DNA composition was first published in *Genetics* in 1974 [4, 5]. 

*C. elegans* measure approximately 1 mm in length in adulthood, have a reproductive life cycle of approximately 3 ½ days when raised at a temperature of 20°C, and are easy to obtain in large populations [6-8]. Animals are maintained in solid or liquid medium, and are typically raised at a temperature range of 15°C to 25°C. The worms can be frozen for long-term storage and remain viable upon thaw. Although worms are self-fertilizing hermaphrodites, males occasionally appear and can be used to transfer genetic markers upon crossing. 

When a bacterial food source is abundant, worms develop through four larval stages (L1-L4) into adulthood. Under conditions of starvation, *C. elegans* enter an alternate stage of development at the second larval molt called dauer and can survive in this arrested state at least four to eight times its normal lifespan [9-11]. Upon introduction to food, dauer larvae resume their developmental progression to adulthood. 

All embryonic and postembryonic cell lineages have been traced for *C. elegans*. Patterns of cell division, migration, and programmed cell death are remarkably constant, and gives rise to a total of 959 somatic cells in the adult hermaphrodite [12-14]. Of these, 302 have been classified as neural cells whose structure and connectivity has also been thoroughly investigated [15]. In addition, the entire *C. elegans* genome has been sequenced, further contributing to the powerful knowledge infrastructure that has been built on this model organism [16].

**Fig. (1):**
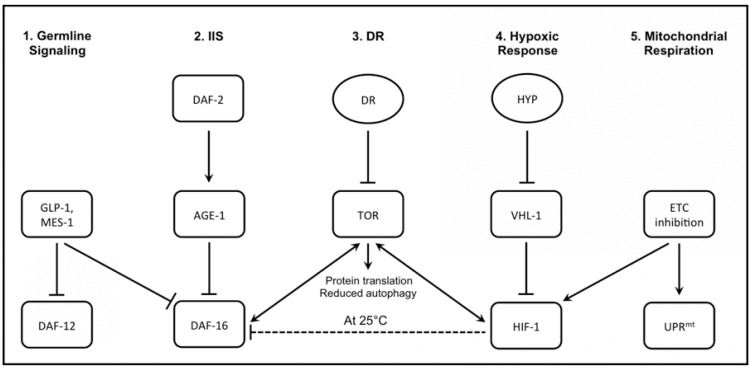
**Simplified model of signaling pathways that influence lifespan in *C. elegans***. Genetic factors (rectangles) and environmental factors (ovals) that are found to affect lifespan have been grouped into several major longevity pathways. (1) Within the germline signaling pathway, mutation of *mes-1* or *glp-1* prevents development of germ cells and results in lifespan extension. This lifespan extension is dependent on both DAF-16 and DAF-12, a putative hormone receptor. (2) The canonical insulin/IGF-1-like signaling (IIS) pathway involves DAF- 2, an insulin-like receptor that promotes activity of the AGE-1 phosphatidylinositol-3-hyroxy kinase, which in turn antagonizes the activity of DAF-16, a FOXO-family transcription factor. Reduction of DAF-2 activity thus activates the longevity-promoting effects of DAF-16. (3) The dietary restriction (DR) pathway has been reported to act independently of IIS. Recent studies suggest that DR inhibits TOR activity, and thereby reduces protein translation and promotes autophagy in *C. elegans*. This model has been proposed as a possible mechanism for the lifespan-extending effects of DR. Interestingly, crosstalk exists between TOR signaling and components of other signaling pathways, such as interactions with DAF-16 or HIF-1. (4) Exposure to hypoxic conditions results in inhibition of VHL-1-dependent ubiquitination of HIF-1 and the subsequent stabilization of HIF-1 to promote lifespan extension. Deletion of *hif-1*, however, has also been found to have a temperaturedependent effect on lifespan, with *hif-1* mutants being long-lived at 25°C but showing similar lifespan to wild-type worms at 15°C or 20°C. Temperature-dependent lifespan extension by deletion of *hif-1* is dependent on DAF-16 under some conditions and may act via TOR-signaling under other conditions (dotted lines). (5) Mutation or RNAi knockdown of genes encoding components of the mitochondrial electron transport chain (ETC) can promote longevity by two different mechanisms: activating the mitochondrial unfolded protein response (UPR^mt^) and stabilization of HIF-1.

Studies that laid the groundwork for aging research in *C. elegans* began nearly thirty years ago, when seminal research by Michael Klass, Tom Johnson, and others employed mutagenesis techniques to isolate long-lived mutants; this led to the formal naming and description of the first *C. elegans *longevity gene *age-1* [17, 18]. A decade later, a second wave of research, spurred by the development of new methods and technologies, pushed beyond the identification of individual longevity genes and began to describe genetic and molecular pathways that regulate aging. It was first discovered that mutation of *daf-2*, a gene involved in repressing dauer formation, could extend lifespan by more than twofold, and that this lifespan extension was dependent on *daf-16 *[19, 20]. Subsequently, mutation of *age-1* was shown to similarly require *daf-16 *for lifespan extension, suggesting that *daf-2 *and *age-1 *function within a single genetic pathway [21]. Soon after this, the molecular functions of *daf-2*, *age-1*, and *daf-16 *were uncovered; DAF-2 functions as an insulin-like receptor that promotes the activity of the AGE-1 phosphatidylinositol-3-hydroxy kinase that antagonizes the activity of DAF-16, a FOXO-family transcription factor [22-25]. Together, these factors defined an insulin/IGF-1-like signaling (IIS) pathway that modulates longevity in *C. elegans *(Fig. **1**). 

Based on studies of the IIS pathway, DAF-16-dependence or independence has come to serve as the most utilized genetic test for grouping longevity factors. Mutations that increase lifespan in a DAF-16-dependent manner are generally referred to as acting within the IIS pathway, although it is now clear that multiple factors can modulate DAF-16 activity by mechanisms that are both similar and different from reduced *daf-2 *activity [3]. For example, several studies have identified a signal from the germline that limits longevity in a DAF-16-dependent manner [3]. Laser ablation of the germline results in lifespan extension that is requires both *daf-16* and *daf-12*, a putative hormone receptor [26]. Researchers observed similar lifespan extension in *mes-1* and *glp-1* mutants, both lacking germ cells. Furthermore, lifespan extension in *mes-1* or *glp-1* mutants is suppressed by *daf-16* null mutation or by ablation of somatic gonad precursor cells. Specifically, the absence of undifferentiated germ-line stem cells during development or adulthood was shown to result in lifespan extension [27]. 

Several genetic factors that modulate lifespan independently of DAF-16 have been characterized in detail: mimetics of dietary restriction (DR), mutations in mitochondrial electron transport chain (ETC) enzymes, and mutations that activate the hypoxic response. These include the loss of function alleles of the mitochondrial coenzyme Q biosynthetic enzyme CLK-1 [28-30], a series of *eat *mutations that reduce pharyngeal pumping and food consumption [31], and mutations that stabilize the hypoxic response transcription factor, HIF-1 [32-35]. Further studies have suggested that each of these classes of longevity factors act within distinct epistasis groups, leading to the current model for at least four distinct longevity pathways in *C. elegans *(Fig. **1**).

However, this four pathway model is complicated by evidence of known sites of crosstalk between these pathways. For example, the target of rapamycin (TOR) kinase interacts with IIS, DR, and HIF-1 [36-39]. As another example, some (but not all) mutations that impair mitochondrial function and extend lifespan also activate HIF-1 and require HIF-1 for lifespan extension [40]. Thus, it is clear that these genetically distinct pathways are actually components of a complex longevity network. Whether this network will ultimately be found to converge on similar downstream mechanisms for longevity control remains to be determined. 

### A Cautionary Note on Methodology

One of the unique features about *C. elegans *as a model organism is that the vast majority of studies are performed in a common genetic “wild type” background referred to as N2. Thus, it is often assumed that experimental methodology among different labs is relatively constant. However, recent controversies have demonstrated that even seemingly subtle differences in methods used for longevity studies can have substantial effects on outcome. Three important methodological components that can influence lifespan are (1) the use of live versus dead *E. coli *as a food source, (2) the temperature at which the experiments are performed, and (3) the use of 5-fluorodeoxyuridine (FUdR) to prevent hatching of progeny.

A majority of *C. elegans *labs use the *E. coli *strain OP50 as a food source. The bacteria are cultured on the surface of a nutrient-agar Nematode Growth Medium (NGM), and the animals crawl through the bacterial lawn to feed [41]. Several years ago, it was discovered that simply killing the *E. coli *food via UV-radiation or antibiotic treatment was sufficient to increase mean and maximum lifespan of *C. elegans* [42], and a similar effect was found when a non-pathogenic *Bacillus subtilis *food source was used [43]. This has led to the model that bacterial pathogenesis limits the survival of the population when live bacterial food is used. Interestingly, several long-lived mutants, including components of the IIS pathway, are resistant to known bacterial pathogens [43, 44], raising the possibility that at least a portion of the lifespan extension in these cases results not from a direct effect on aging but instead from reduced death due to infection. For this reason, some groups (including ours) choose to use killed bacterial food for a majority of lifespan experiments, with the exception of RNAi experiments (see below for details) [41]. One consequence of this is that some interventions that appear to extend lifespan on live *E. coli* may no longer extend lifespan on killed *E. coli* if their mechanism of action involves reducing death due to bacterial pathogenesis.

Temperature was one of the first major determinants of *C. elegans *longevity to be identified. Similar to the case in fruit flies [45], lifespan decreases as temperature increases, within the range of about 10°C-30°C [46]. The most common temperature used for studies of *C. elegans* aging is 20°C; however, many groups use 25°C, and a few use 15°C. It has been generally assumed that an intervention that extends or shortens lifespan at one temperature will have a proportionately similar effect on lifespan at another temperature, but there is little experimental data to support this assumption. Instead, recent studies of HIF-1 have demonstrated that, at least for this longevity factor, temperature is a key determinant of whether deletion of *hif-1 *shortens or lengthens lifespan [47]. At 25°C, animals carrying the *hif-1(ia4) *null allele are long-lived, while at 15°C they have a lifespan that is not significantly different from wild type N2 animals [32, 38]. Contradictory results have been reported for experiments performed at 20°C, with some studies showing lifespan extension and others detecting no effect on lifespan [32-35, 48]. These conflicting reports initially resulted in confusion, until the temperature-dependent nature of the *hif-1(ia4) *longevity phenotype was uncovered [49]. Interestingly, stabilization of HIF-1 by deletion of the gene coding for the VHL-1 E3 ligase extends lifespan throughout the temperature range from 15-25°C [32]. Thus, temperature plays a critical role in determining the longevity effect of *hif-1 *deletion but has little, if any, effect on the lifespan extension from HIF-1 stabilization (Fig. **1**). 

The chemical FUdR has become widely used in *C. elegans *aging studies, in order to prevent hatching of progeny during lifespan experiments [41, 50]. If FUdR is not used, experimental animals must be transferred to fresh NGM plates every 24 to 48 hours during the first 7 to 14 days of the experiment. In contrast, when FUdR is added to the NGM beginning at L4, animals will become sterile and need only be transferred to new plates when the food supply becomes limiting. Several groups have reported that at concentrations used for aging studies (50-100 *μ* g/ml), FUdR does not alter the longevity of wild type N2 animals [51]. However, recent work suggests that some genes influence longevity differently depending on whether FUdR is present or not. For example, FUdR has been found to significantly increase lifespan in mutants deficient in *tub-1*, a gene involved in modulation of fat storage, and a similar lifespan-extending effect has been reported for the mitochondrial mutant *gas-1* [52, 53]. The effects of knock-down of genes that modulate histone H3 lysine 4 trimethylation also vary depending on whether animals are sterile or fertile [54]. Thus, *C. elegans* aging studies that utilize FUdR may yield results that are different from studies performed in the absence of FUdR, as the chemical itself may be acting on specific genes and genetic pathways under investigation. 

### Methods and Mechanisms of Dietary Restriction

DR, which has been described as nutrient restriction without malnutrition, is without question the most widely studied longevity intervention. There are reports of lifespan extension from DR in many different species, including yeast, nematodes, fruit flies, mice, rats, dogs, fish, spiders, and monkeys [55-57].

In addition to *eat-2* mutation, a number of methods for DR have been developed for *C. elegans* utilizing solid and liquid mediums, and each method has been shown to be effective in increasing lifespan [38, 46, 58-62]. However, a complete understanding of the mechanism by which DR promotes longevity is complicated by results showing that different DR methods extend lifespan by both independent and overlapping genetic pathways [63, 64]. Whereas DR by bacterial deprivation on solid medium, *eat-2* mutation, or axenic liquid medium extends lifespan independent of *daf-16*, lifespan extension resulting from bacterial dilution in liquid medium is at least partially dependent on *daf-16*. Thus, individual genes that have been identified to act within the DR longevity pathway may be specific to the DR method under study. 

Interestingly, mutations that cause defects in sensory perception in *C. elegans* results in *daf-16*-dependent lifespan extension, suggesting that longevity is partially regulated by environmental cues [65]. This hypothesis is further supported by research showing that laser ablation of specific sensory neurons, individually or in combination, has also been found to extend lifespan [66]. Food sensing has also been shown to play a role in lifespan extension from DR that is independent of *daf-16 *[67], suggesting that sensory perception can modulate lifespan by multiple mechanisms.

Recently, the possible involvement of the TOR pathway in DR has generated considerable interest in the aging field. The TOR pathway is a highly conserved pathway that has been implicated in regulation of metabolic processes and aging in both invertebrate and mammalian models [68, 69]. Molecular components of TOR signaling were originally identified through yeast studies involving rapamycin, a drug that is known to specifically inhibit TOR complex 1 (TORC1) signaling. [70]. TORC1 consists of TOR kinase partnered with Raptor, PRAS40 (proline-rich AKT substrate 40 kDa), and a G *β* L protein [71]. Inhibition of the TORC1 results in decreased protein translation, through reduced phosphorylation of S6 kinase (S6K) or activation of 4E-binding protein (4EBP), an inhibitor of eukaryotic translation initiation factor 4E (eIF4E) [72, 73]. 

Several lines of evidence suggest that TORC1 signaling plays an important role in lifespan extension from DR in different organisms; genetic or pharmacological inhibition of TORC1 is sufficient to extend lifespan in yeast, nematodes, flies, and mice, and in yeast, nematodes, and flies, inhibition of TORC1 fails to increase lifespan additively with DR [74]. In *C. elegans*, reduced mRNA translation and induction of autophagy downstream of TORC1 appear to be important for lifespan extension [75, 76]. 

### Genome-Wide RNAi Methods in *C. elegans*

Before RNAi technology was developed, aging research in *C. elegans *was largely limited to “forward” genetic screens in which random mutations were identified, lifespan quantified, and in some cases the gene was identified through classical methods. The discovery of gene knockdown by RNA interference (RNAi) in *C. elegans *paved the way for a revolution in the pace of aging research. Initially, injection of double-stranded RNA was found to be an effective method of genetic interference [77]. Soon afterwards, it was found that soaking worms in dsRNA solution or feeding worms bacteria engineered to produce dsRNA could also induce RNAi knockdown [78, 79], and that feeding was as equally as effective as injection methods [80]. Development of the bacterial feeding RNAi method led to construction of genome-wide RNAi libraries, which have become extremely useful tools for *C. elegans* research. The Ahringer library contains 16,757 clones, which were generated by cloning gene-specific genomic fragments between two inverted T7 promoters [81, 82]. The Vidal library was more recently developed and contains 11,511 clones, produced by Gateway cloning of full-length open reading frame (ORF) cDNAs into a double T7 vector [83]. 

The construction of genome-wide RNAi libraries in *C. elegans* provided a more systematic way of studying aging. For the first time, individual genes could be knocked down and the effect on lifespan directly measured for a majority of the *C. elegans *genes. Other aging-associated phenotypes, such as fecundity, developmental rate, and lipofuscin accumulation could be measured on a large scale. To date, hundreds of RNAi screens have been performed in *C. elegans *for a variety of phenotypes, and much of this data is curated on Wormbase [84]. The foremost purpose of this review is to revisit the first large-scale genetic screens that identified a number of functional classes of genes that influence aging and to compare and contrast these studies. We will provide an in-depth analysis of the consistencies and inconsistencies between published results, and highlight differences in methodologies that may have influenced their findings. Lastly, an important aim of this review will be to relate the early publications with successive trends in aging research. 

## INITIAL RNAi SCREENS TO IDENTIFY LONGEVITY GENES AND PATHWAYS

The era of longevity genomics was ushered in with parallel genome-wide RNAi screens for increased lifespan performed by the Ruvkun and Kenyon labs. The first published reports from both labs examined genes on chromosomes I and II and came to the same primary conclusion: knockdown of mitochondrial electron transport chain (ETC) genes by RNAi is sufficient to extend lifespan in *C. elegans* [85, 86]. The Kenyon lab study authored by Dillin *et al. *was published first, and established that reduced ETC function extended lifespan of both *daf-2* and *daf-16* mutant animals, suggesting that mitochondrial respiration controlled longevity independent of the IIS pathway. The RNAi clones with the most robust effect on lifespan were *nuo-2*, *cyc-1*, *cco-1*, and *atp-3* which are subunits of complex I, III, IV, and V, respectively [85]. 

Consistent with these observations, the Ruvkun lab publication authored by Lee *et al.* [86] found that 15% of the RNAi clones that extended lifespan in their screen corresponded to nuclear encoded mitochondrial genes. Similar to Dillin *et al., *this study also found that knockdown of complex I, III, and IV subunits could extend lifespan of both wild type and *daf-16(mgDf47) *animals. Interestingly, Lee *et al. *also identified a handful of mitochondrial RNAi clones, such as mitochondrial carrier gene F13G3.7 and phosphoglycerate mutase gene F57B10.3, that extended lifespan in a DAF-16-dependent manner, thus suggesting interplay between mitochondrial function and IIS with respect to longevity control. In addition, the researchers found that RNAi inactivation of only 20% of known mitochondrial genes tested had a detectable lifespan phenotype. This may be explained by the efficiency of knockdown inherent to different RNAi clones; or perhaps a more intriguing possibility is that distinct types of mitochondrial dysfunction lead to lifespan extension. 

Both labs proceeded to publish follow-up papers describing the comprehensive results of their screens of the entire Ahringer RNAi library [87, 88]. Epistasis analysis was used to place the newly identified longevity genes into pathways and functional categories. The results of these epistasis tests are summarized below (Table **1**). Hansen *et al*. [87] reported 23 additional novel longevity factors along with previously reported longevity genes including *cyc-1*, *cco-1*, *nuo-2*, *atp-3*, *cchl-1* and *daf-2*. To study if the newly identified longevity genes acted within the IIS and germline pathways, the effect of RNAi on lifespan was determined in *daf-16(mu86)* animals and *daf-2(e1370)* animals. For those genes showing DAF-16-dependent lifespan extension, RNAi knockdown was also performed in *glp-1(e2141)* animals that lack a germline when raised at high temperatures and *daf-12(rh61rh411)* animals. GLP-1 and DAF-12 both function in the germline longevity pathway (Fig. **1**) to modulate longevity through DAF-16. The genes *ttr-1, sinh-1, ddl-1, ddl-2, *and* ddl-3 *were identified from this analysis as novel DAF-16-dependent longevity genes that also function, at least partially, in the germline signaling pathway.

In order to determine whether newly identified longevity genes acted by a mechanism similar to DR, Hansen *et al*. [87] combined RNAi knockdown with mutation of *eat-2*. Knockdown of *sams-1* (C49F5.1), *rab-10* (T23H2.5), *drr-1* (F45H10.4), and *drr-2 *(T12D8.2) were unable to further extend the lifespan of *eat-2* animals, suggesting that they function in the DR pathway. In contrast, RNAi knockdown of ETC components further extended the lifespan of *eat-2(ad1116) *animals, supporting the idea that DR and ETC knockdown act by distinct mechanisms. Interestingly, a total of only three genes were unable to be placed into known longevity pathways including two proteins with no obvious homologs, *ril-1 *(C53A5.1) and *ril-2 *(C14C10.3), and the DEAH RNA helicase *rha-2 *(C06E1.10).

The genome-wide RNAi study conducted by the Ruvkun lab, authored by Hamilton *et al.* [88], identified a total of 89 additional aging genes with disparate functions including cell structure, cell surface proteins, cell signaling, cellular metabolism, and protein turnover. Of the 66 genes with previously known functions, 17 corresponded to various aspects of carbon metabolism, including citric acid cycle enzymes and subunits of complexes I, IV, and V of the ETC. Researchers also speculated that protein translation might play a role in lifespan regulation, based on the identification of *iff-1* (T05G5.10), a gene that has homology to the translation initiation factor eIF5A. Other hits from this screen included two genes containing PH domains known to interact with phosphatidylinositol lipids, multiple G protein-coupled receptors, protein processing and degradation genes such as proteases and ubiquitin ligases/hydrolases, and chromatin modifying factors. 

To further investigate genes that directly affect mitochondrial function, Hamilton *et al*. [88] measured the induction of the mitochondrial unfolded protein response (UPR^mt^) using a GFP reporter strain for activation of HSP-6, a mitochondrial chaperone. Impaired assembly of mitochondrial multi-subunit complexes or disruptions in protein folding or processing caused by inactivation of mitochondrial chaperones and proteases has been previously shown to induce this stress response [89]. They determined that RNAi of ETC subunits and aconitase activated the *hsp-6*:: GFP reporter, in addition to genes of unknown function Y53F4B.23 and Y75B8A.33, the tripartite motif-containing protein C39F7.2, and the cadherin homolog *cdh-12*. 

Tests for epistasis placed 35 of the identified genes with the most robust effects on lifespan within IIS or sirtuin longevity pathways. SIR-2.1 is orthologous to the yeast Sir2 protein, which functions as an NAD-dependent histone deacetylase [90]. Overexpression of Sir2 is sufficient to extend lifespan in yeast [91, 92], and overexpression of *sir-2.1 *was reported to also extend lifespan in *C. elegans* [93], although that result has since become controversial [94]. Of the 35 RNAi clones tested, 26 required DAF-16 for lifespan extension, 11 required SIR-2.1, and 9 required both genes. This indicates that SIR-2.1 and DAF-16 likely influence lifespan in *C. elegans *via both overlapping and distinct mechanisms.

The initial genome-wide RNAi screens revealed that knockdown of hundreds of genes can have positive effects on *C. elegans *longevity. Interestingly, the majority of the genes could be placed into canonical longevity pathways including insulin/IGF, germline, TOR signaling, and mitochondrial respiration. Thus, it may be that the majority of signaling pathways that contribute to aging in *C. elegans *have already been discovered. The recurrent identification of mitochondrial genes in each of the screens collectively suggests that attenuating mitochondrial function in *C. elegans* has positive effects on lifespan, despite the effects of lower ATP production, small body size, slow pumping, slow growth rate, and sterility [85, 86]. Initially, researchers hypothesized that decreasing mitochondrial function lowers oxidative damage and thereby promotes longevity. As described in the next section, however, there is not a perfect correlation between resistance to ROS and longevity. Furthermore, the reduction in mitochondrial function must be a partial rather than severe in order to extend lifespan, as shown by RNAi dilution experiments [95]. Recently, the Dillin lab has proposed a model in which induction of UPR^mt^ can promote longevity [96]. RNAi of several ETC components and some mitochondrial genes shown to increase lifespan, also induce this response [88, 89]. Thus, an intriguing possibility is that UPR^mt^ induction may better protect *C. elegans* from protein misfolding and aggregation events that occur with age.

## COMPARISON OF GENOME-WIDE LONGEVITY SCREENS AND METHODOLOGIES USED

Together, the initial genome-wide longevity screens performed by the Kenyon and Ruvkun labs identified approximately 120 RNAi clones that significantly increased lifespan (Table **1**). Although similar functional groups were identified in each screen, somewhat surprisingly, only 4 RNAi clones were found to extend lifespan by both labs, corresponding to *cchl-1 *(T06D8.6), *cco-1 *(F26E4.9), *drr-1* (F45H10.4), and *nuo-4* (K04G7.4) [85-88, 97]. Several factors may have contributed to this lack of replication, including differences in temperature, strain backgrounds, use of FUdR, and variability in knockdown efficiency (Table **2**).

The initial genome-wide longevity RNAi screens were designed to identify RNAi clones that increase maximal lifespan. This is a straightforward method, as an increase in maximal lifespan is detected by survival of a subset of the RNAi-treated cohort after all temperature-matched control worms have died. One potential disadvantage to this approach is that maximal lifespan extension does not always reflect changes in mean or median lifespan. Hansen *et al.* specifically tested a subset of genes identified in the Ruvkun lab’s Chromosome I/II screen to see if they could replicate their results and observed that the RNAi clones increased mean lifespan, but not maximal lifespan, under their conditions. 

Temperature has a dramatic effect on *C. elegans* lifespan, with increasing temperature causing decreased lifespan in N2 animals [46]. There is limited information on the interaction between genotype and temperature with respect to lifespan, but it may be that some RNAi clones extend lifespan in a temperature-dependent manner. In the case of Hansen *et al*. [87], their lifespan screen was performed at 20°C or 25°C for each chromosome gene set, whereas Hamilton *et al.* [88] carried out all lifespan assays at 25°C. 

Rather than having to separate adult worms from their progeny on a daily basis, different methods to prevent reproduction were taken by each group. Hansen *et al.* [87] grew the CF512 (*fer-15(b26);fem-1(hc17)*) temperature sterile strain at 25°C and propagated worms at 25 or 20°C on RNAi bacteria. In contrast, Hamilton *et al*. [88] used FUDR added during adulthood to prevent eggs from hatching; although N2 worms were used for the initial screen, the RNAi sensitized *rrf-3(pk1426)* strain was used for follow-up validations. Together these differences in experimental approach may have led to identification of different RNAi clones in each screen. It is also important to note that for a majority of the longevity genes identified in each screen, there has been limited (if any) further analysis. Thus, it is currently not possible to estimate the rate of false positive error present in each dataset. In contrast, additional studies (described below) have identified dozens of new longevity factors not identified in the initial genome-wide RNAi screens, suggesting a significant number of false negative identifications, even when the genome-wide screens are combined. 

## POST-GENOMIC LONGEVITY RNAi SCREENS IDENTIFY ADDITIONAL AGING GENES 

Since the publication of the first genome-wide screens for longevity, RNAi technology has been used to identify additional genes that regulate aging and investigate potential aging mechanisms. One important caveat of the four original longevity screens is that RNAi knockdown was initiated either at hatching or at the L1 stage of development (Table **2**). Since many RNAi clones result in larval arrest and other developmental defects, the effect of these genes on adult longevity can not be assessed using such an approach. 

In order to address this limitation, two studies screened essential genes for effects on lifespan by initiating RNAi knockdown at the L4 stage of development. Chen *et al.* [98] tested 57 RNAi clones that had previously been identified as causing larval arrest phenotypes as a result of RNAi inactivation [81]. In their screen, RNAi treatment was initiated at the L4 stage of development so as not to adversely affect development at early embryonic and larval stages. Of the 57 genes that were screened, RNAi knockdown of 24 clones was found to extend lifespan significantly in wild-type *C. elegans*. Most of these genes have been implicated in regulation of mRNA translation and mitochondrial functions. RNAi knockdown of all the identified genes further extended lifespan of both *daf-2* mutants and *daf-16;daf-2* double mutants, leading researchers to conclude that these genes are likely to affect lifespan independently of IIS. In addition, the lifespan-extending RNAi clones were also assayed for effects on stress-resistance and fecundity. All of the RNAi clones tested, except for one, reduced fecundity significantly compared to wild-type. Knockdown of most of these genes also resulted in increased resistance to heat stress, while knockdown of ~50% of these genes resulted in increased resistance to juglone, a compound that induces oxidative stress. 

A similar study by Curran and Ruvkun [99] investigated a broader set of RNAi clones, screening knockdown of 2,700 genes essential for development. Post-developmental RNAi knockdown of 64 (~2.4%) of these genes was found to extend lifespan, a 4-fold increase in frequency of lifespan extension relative to their prior genome-wide screen (89/16,000, ~0.6%) [88]. Major functional classes of genes uncovered in this study include those involved in protein synthesis, RNA binding/processing and chromatin-associated factors, signaling molecules, and mitochondrial function. Lifespan extension for many of the identified genes, most notably those that encode signaling molecules and RNA-related genes, was dependent on *daf-16*, suggesting that these genes act within the IIS pathway to regulate longevity. Other gene inactivations, particularly those targeting protein synthesis machinery and mitochondrial function, appeared to extend lifespan independent of *daf-16. *However, many of the RNAi clones that were not dependent on *daf-16* for lifespan extension still induced nuclear localization of *daf-16* and expression of its target *sod-3*, as visualized in transgenic GFP animals; this was especially true for genes involved in protein synthesis. 

Comparison of these two screens of genes required for development [98, 99] reveals only four genes in common – *eIF-3f *(D2013.7), F59C6.5, *spg-7 *(Y47G6A.10) and *atp-3 *(F27C1.7) – and technical considerations may partially explain the lack of overlap. Chen *et al.* tested RNAi knockdown of essential genes in wild-type (N2) animals, whereas Curran and Ruvkun utilized the RNAi sensitive *eri-1(mg366)* strain. Furthermore, Chen *et al.* tested a very small subset of 57 essential genes, whereas Curran and Ruvkun tested 2,700. The results of epistasis experiments relating to the IIS pathway also showed some inconsistencies between studies; whereas Chen *et al.* found that all lifespan extending RNAi clones from their screen could extend lifespan independent of *daf-16*, Curran and Ruvkun found many genes appear to act within the IIS pathway to regulate longevity. However, as with the different genome-wide RNAi screens, both studies yielded similar functional classes of genes. Notably, both RNAi screens revealed longevity genes that are involved in mitochondrial function, including two genes, F26E4.6 and *atp-3*, that had been identified to increase lifespan in previous genome-wide screens [88]; however, prior research had indicated that lifespan extension resulting from inactivation of mitochondrial genes required initiation of RNAi treatment during larval development [85]. Both groups speculated that the discrepancy might be explained by the variable effectiveness of RNAi. Curran and Ruvkun utilized an RNAi-sensitive strain in which post-developmental knockdown of mitochondrial components might be effective, and Chen *et al.* identified mitochondrial RNAi clones with strong larval arrest phenotypes in wild-type worms. It may also be that the novel mitochondrial genes from these studies extend lifespan by a mechanism that is different from the mitochondrial genes that were examined in previous screens.

Among the essential genes that influence aging, one particularly important class are factors that are involved in cytoplasmic mRNA translation [100]. Curran and Ruvkun identified 10 genes that promote mRNA translation and protein synthesis; several of these RNAi clones targeted components of the eukaryotic translation initiation factor complex (eIF4F) and increased lifespan up to 50% longer than with vector control RNAi. Chen *et al.* identified 10/24 genes involved in translation initiation as well as other components of translational machinery such as ribosomal biogenesis and tRNA synthesis. The involvement of protein synthesis and translation in aging has been further validated by studies from the Kenyon, Tavernarakis, and Kapahi labs, demonstrating that reduction in mRNA translation is often associated with increased lifespan in *C. elegans* [76, 101, 102]. RNAi of multiple ribosomal protein genes and translation initiation factors can extend lifespan, as can mutant alleles of ribosomal-protein S6 kinase (S6K*/rsks-1*) and the translation initiation factors eIF4G (*igf-1*), and eIF4E (*ife-2*).

## IDENTIFYING LONGEVITY GENES USING CORRELATED PHENOTYPES

Rather than use extended lifespan as the primary phenotype, some groups have screened for alternative phenotypes, such as altered stress resistance, prior to testing for effects on longevity. The list of such studies is extensive and beyond the scope of this review; however, two representative examples are described in detail here. 

Kim and Sun [103] sought to explore the relationship between ROS sensitivity and lifespan in *C. elegans* by performing an extensive screen to identify RNAi clones that provided resistance to the ROS-generating compound paraquat, and then screened positive hits for effects on longevity. A total of 84 RNAi clones were identified that conferred paraquat resistance and greater than 10% lifespan extension. Epistasis experiments revealed that 29/52 non-mitochondrial hits from this screen were *daf-16*-dependent. These genes include proteins with disparate functions including the transport of nutrients, stress responses, chromatin remodeling/gene expression, protein-protein interactions, and protein turnover. Among the *daf-16*-independent longevity genes were *pat-4 *(C29F9.7), which encodes the integrin-linked kinase (ILK) that was also identified in an independent screen [87]. Some RNAi clones differentially increased lifespan of *daf-16* null mutants and wild-type animals, such as ribosome components *rps-23 *(F28D1.7) and F33D4.5, suggesting a partial interaction with IIS. 

Interestingly, the majority of RNAi clones that enhanced paraquat resistance did not extend lifespan [103]. This supports the idea that resistance to ROS is not sufficient to increase lifespan. Furthermore, approximately 30% of the hits from this screen are annotated as mitochondrial proteins, such as respiratory chain components, mitochondrial ribosomes, and ADP/ATP carriers. These results are consistent with a model in which superoxide reaction caused by paraquat requires electrons derived from the respiratory chain; however, the majority of reported mitochondrial RNAi clones that extend lifespan also cause decreased resistance to paraquat [86]. Of the longevity genes identified in this study, only 4 – *pat-4 *(C29F9.7), *nuo-3 *(Y57G11C.12), *nuo-4 *(K04G7.4), and T20H4.5, a NADH-quinone oxidoreductase, – were previously identified by the initial genome-wide RNAi screens.

Mehta *et al*., [33] screened RNAi clones related to proteasome function for enhanced resistance to polyglutamine toxicity, under the assumption that enhanced resistance to proteotoxic stress is often an indicator of longevity in *C. elegans*. For this screen, a strain expressing a 35 glutamine repeat fused to GFP in body wall muscle cells was employed [104]. Animals expressing this transgene show an age-dependent increase in aggregation that results in paralysis, without a significant reduction in lifespan [105]. Not surprisingly, *Mehta et al.* 33] found that a majority of RNAi clones led to enhanced toxicity and shortened lifespan; however, knockdown of the E3 ligase gene *vhl-1* resulted in resistance to polyglutamine toxicity and increased lifespan. VHL-1 is the* C. elegans *homolog of the Von Hippel-Lindau tumor suppressor gene and functions as a negative regulator of HIF-1. Mehta *et al*. also observed the RNAi knock-down of the EGL-9 prolyl hydroxylase gene, which functions upstream of VHL-1 to promote degradation of HIF-1, also enhances resistance to polyglutamine toxicity and increases life span. Several other groups have since confirmed that stabilization of HIF-1 is sufficient to extend lifespan in worms by a mechanism distinct from IIS or DR, establishing this as a major longevity pathway [34, 35, 48]. Interestingly, this pathway also was not identified from the genome-wide longevity RNAi screens, further illustrating the point that many longevity genes still remain to be defined. 

## CONCLUSION

Studies utilizing RNAi technology to identify genes and pathways that regulate the aging process in *C. elegans *have yielded an abundance of useful and interesting results. They have helped define the most important pathways and processes modulating longevity in *C. elegans*, and have served to promote the search for conserved longevity factors in other organisms [106]. Although there is consensus that mitochondrial function, mRNA translation, and IIS are key determinants of longevity, the degree of overlap across studies at the individual gene level is surprisingly small. Many factors may contribute to this lack of replication, including important differences in experimental methods and variable effectiveness and specificity of RNAi clones. With more than 300 different RNAi clones already reported to extend lifespan, it remains unknown how many more remain to be identified. Future studies will undoubtly continue to uncover new longevity genes and mechanisms to provide a deeper understanding of the biology of aging. 

## Figures and Tables

**Table 1.** Epistasis Relationships for Longevity Genes. A Limited Set of Epistasis Studies have been Performed in Conjunction with Different RNAi Screens to Characterize Novel Longevity Genes. Data are shown for Experiments Combining RNAi Knock-down of the Longevity Genes in Combination with Mutation of *daf-16, glp-1, eat-2, or sir-2.1*. Dependence Indicates that the Factor is Required for Further Life Span Extension from the RNAi Clone (e.g. DAF-16 Dependence means the RNAi Clone Fails to Extend Life Span in a *daf-16* Mutant Background). Genes Annotated As Mitochondrial are Listed in the “Mitochondrial” Group. Gene Names are Annotated According to Wormbase. Superscript Numbers Represent the Study in which the RNAi Clone was Identified. ^1^Chen, D., *et al*., Aging Cell, 2007, ^2^Curran, S.P. and G. Ruvkun, PLoS Genet, 2007, ^3^Dillin, A., *et al*., Science, 2002, ^4^Hamilton, B., *et al*., Genes Dev, 2005, ^5^Hansen, M., *et al*., PLoS Genet, 2005, ^6^Hansen, M., *et al*., Aging Cell, 2007, ^7^Kim, Y. and H. Sun, Aging Cell, 2007, ^8^Lee, S.S., *et al*., Nat Genet, 2003, ^9^Pan, K.Z., *et al*., Aging Cell, 2007

DAF-16
Dependent
* aat-8*^7^,* acs-5*^7^,* age-1*^4^,* akt-1*^4^,* amt-2*^7^,* asm-3*^7^,* atp-2*^2^,**B0546.3^7^, C09B7.2^4^, C32H11.1^4^, C36H8.1^4^, C39E9.1^4^, C42C1.3^7^, C46G7.2^7^, C56G2.1^2^, *cct-6*^2^,* ced-3*^2^,* col-93*^4^,* cpna-3*^4^,* crn-5*^2^,* cutl-28*^7^,* cwp-4*^4^,**D1054.14^2^, *daf-2*^5,7,2^,* ddl-1*^5^,* ddl-2*^5^,* ddl-3*^5^,* eIF2b/iftb-1*^6^,* eIF4G/ifg-1*^6^,* epc-1*^7^,* erm-1*^2^,* ero-1*^2^,**F09C11.1^7^, F13G3.7^8^, F19B6.1^2^ , F21H12.1^4^, F26A3.4^2^, F28B3.5^8^, F42A6.1^7^, F49C12.9^7^, F53F4.11^2^, F55B11.1^4^, F56D5.5^7^, F57B10.3^8^, *gei-15*^2^,* glp-1*^2^,* gpi-1*^5^,* grl-19*^7^,* hrp-1*^2^,* inx-14*^4^,* inx-8*^7^,* inx-9*^7,2^,**K07H8.8^7^, *maoc-1*^5^,* mcm-2*^2^,* mrpl-50*^2^,* nol-1*^2^,* npa-1*^2^,* nuo-1*^2^,* pod-1*^7^,* pos-1*^2^,* prx-5*^2^,**R05A10.5^4^, R10H10.7^7^, *ral-1*^7^,* sas-5*^2^,* scrm-8*^4^,* sel-5*^2^,* sem-5*^2^,* set-9*^4^,* sinh-1*^5^,* spe-26 *^7^,* spg-7*^2^,* sre-25*^2^,* str-49*^2^,**T05A1.5^7^, T05G5.10^4^, T06G6.4^4^, T07A9.8^2^, *tag-300*^2^,* tpa-1*^2^,* trim-9*^4^,* ttr-1*^5^,* ttr-5*^7^,* unc-52*^2^,**W07G9.1^7^, *wdr-23*^2^,* wts-1*^2^,**Y105C5B.12^2^, Y119D3_446.d^7^, Y39F10C.1^4^, Y39H10A.6^4^, Y43h11AL.2^4^, Y45F10D.8^7^, Y46H3C.1/.2^4^, Y51H4A.m^7^, Y54E10BR.4^2^, Y56A3A.19^2^, Y56A3A.9^4^, Y65B4BR.5^2^, Y71H2_390.d^7^, Y71H2AR.2^4^, Y75B8A.13^4^, Y75B8A.33^4^, ZC132.3^2^, ZK1127.5^2^, ZK686.2^2^
** Dependent on GLP-1; Germline Signaling**
* ddl-1*^5^,* ddl-2*^5^,* ddl-3*^5^,* sinh-1*^5^,* ttr-1*^5^
** Independent**
* abcx-1*^1^,* acdh-13*^7^,* aco-2*^4^,* asb-2*^5^,* atp-3*^3,5,2^,* atp-4*^5^,* atp-5*^5^,**B0491.5^1^, B0511.6^2^, C48E7.2^1^, *cchl-1*^5,8^,* cco-1*^3,5,8^,* cco-2*^5^,* cct-4*^2^,* ceh-18*^4^,* cyc-1*^3,5^,**D2030.4^8^, D2085.3^1^, *dic-1*^2^,* drr-1*^5^,* drr-2*^5^,* egl-45*^2^,* eif-1*^2^,* eif-3.B*^2^,* eif-3.F*^2,1^,* eIF4G/ifg-1*^9^,* elt-6*^7^,* exos-3*^1^,**F08B4.7^7^, F09F7.5^4^, F13B6.1^7^, F14B4.3^1^, F26E4.4^1^, F26E4.6^8,2^, F33D4.5^7^, F59C6.5^1,2^, *hhat-2*^7^,* htp-3*^2^,* ifg-1*^2^,* inf-1*^2^,**K01C8.7^8^, K08E3.5^4^, K11B4.1^1^, K12H4.5^7^, LLC1.3^7^, *lpd-5*^1^,* mars-1*^7^,* mrpl-10*^1^,* mrpl-24*^1^,* mrpl-47*^8^,* mrps-30*^1^,* mtch-1*^2^,* nars-1*^1^,* nol-5*^1^,* nuo-2*^3,5^,* nuo-3*^5^,* nuo-4*^4,5^,* nuo-5*^5^,* pat-4*^5,7,2^,* pat-6*^5^,* phi-37*^2^,* qars-1*^7^,**R08E3.3^4^, R53.4 ^1^, *rab-10*^5^,* rha-2*^5^,* ril-1*^5^,* ril-2*^5^,* rpl-19*^1^,^6^,* rpl-4*^6^,* rps-11*^2^,* rps-15*^6^,* rps-22*^6^,* rps-23*^7^,* rps-3*^2^,* rps-5*^7^,* rps-8*^2^,* S6K/rsks-1*^6,9^,* sams-1*^5^,* scl-8*^7^,* sco-1*^1^,* spg-7*^1^,* spt-4*^4^,* symk-1*^2^,**T02H6.11^8^, T20H4.5^4^, T22B11.5^7^, T28A8.6^7^, T28D6.4^7^, *tars-1*^1^,* tes-1*^7^,* tkt-1*^7^,* TOR/let-363*^6^,* unc-62*^2^,* vha-6*^2^,**W03G1.5^7^, W09C5.8^8^, Y39G10AR.8^1^, Y48G1A.4^1^, Y54E5A.7^4^, Y66A7A1^7^, Y69A2A_2991.c^7^, Y71H2_388.c^7^
** EAT-2**
**Dependent**
*drr-1*^5^,* drr-2*^5^,* pat-4*^5^,* rab-10*^5^,* sams-1*^5^,* TOR/let-363*^6^
**Independent**
*abcx-1*^1^,**B0491.5^1^, C48E7.2^1^, D2085.3^1^, * eIF2b/iftb-1*^6^,* eif3.F*^1^,* eIF4G/ifg-1*^6,9^,* exos-3*^1^, F14B4.3^1^, F26E4.4^1^, F59C6.5^1^, K11B4.1^1^, *lpd-5*^1^,* mrpl-10*^1^,* mrpl-24*^1^,* mrps-30*^1^,* nars-1*^1^,* nol-5*^1^, * pat-6*^5^,**R53.4 ^1^, *rha-2*^5^,* ril-1*^5^,* ril-2*^5^,* rpl-19*^1^,* rps-15*^6^,* rps-22*^6^,* S6K/rsks-1*^6,9^,* sco-1*^1^,* spg-7*^1^,* tars-1*^1^,**Y39G10AR.8^1^, Y48G1A.4^1^
**SIR2.1**
**Dependent**
C36H8.1^4^, C39E9.1^4^, *ceh-18*^4^,* cpna-3*^4^, F09F7.5^4^, F21H12.1^4^, F55B11.1^4^, R05A10.5^4^, * scrm-8*^4^, Y71H2AR.2^4^, Y75B8A.13^4^
**Independent**
*aco-2*^4^,* age-1*^4^,* akt-1*^4^, C09B7.2^4^, C32H11.1^4^, *col-93*^4^,* cwp-4*^4^,* eIF2b/iftb-1*^6^,* eIF4G/ifg-1*^9^,* inx-14*^4^,**K08E3.5^4^, *nuo-4*^4^,**R08E3.3^4^, *rps-15*^6^,* S6K/rsks-1*^6,9^,* set-9*^4^,* spt-4*^4^, T05G5.10^4^, T06G6.4^4^, T20H4.5^4^, *TOR/let-363*^6^,* trim-9*^4^, Y39F10C.1^4^, Y39H10A.6^4^, Y43h11AL.2^4^, Y46H3C.1/.2^4^, Y54E5A.7^4^, Y56A3A.9^4^, Y75B8A.33^4^
**Mitochondrial**
*aco-2*^4^,* ant-1.1*^7^,* asb-2*^5^,* asg-2*^4^,* atp-2*^2^,* atp-3*^3^,^5^,^2^,* atp-4*^5^,* atp-5*^5^, C33A12.1^7^, C33F10.12^4^, C47E12.2^7^, *cchl-1*^5,8^,* cco-1*^3,4,5,8^,* cco-2*^5^,* cyc-1*^3^,^5^,* cyc-2.1*^7^,* cyp-33E2*^7^,**D2030.4^4,8^, F13G3.7^8^, F26E4.6^4,8,2^, F28B3.5^8^, F29C4.2^7^, F43G9.1^4^, F55B11.1^4^, F57B10.3^4,8^, F59C6.5^1,2^, *idh-1*^4^,**K01C8.7^8^, K12H4.5^7^, *lpd-5*^1^,* mrpl-10*^1^,* mrpl-12*^7^,* mrpl-24*^1^,* mrpl-47*^4^,^8^,* mrps-30*^1^,* mrps-33*^7^,* mrps-9*^7^,* mtch-1*^2^,* nduf-2.2*^7^,* nuo-1*^2^,* nuo-2*^3,5^,* nuo-3*^5^,* nuo-4*^4,5,7^,* nuo-5*^5^,* phi-37*^2^,* qars-1*^7^,**R53.4 ^1^, *sco-1*^1^,* spg-7*^1,2^,**T02H6.11^8^, T20H4.5^4^,^7^, T22B11.5^7^, *tag-174*^7^,* ucr-1*^7^,**W09C5.8^4,8^, Y119D3_463.b^7^, Y37D8A18^7^, Y53G8A_9248.c^7^, Y53G8A_9248.d^7^, Y55F3B_743.b^7^, Y56A3A.19^7,2^, Y57G11C.12^7^, Y71H2_378.a^7^, Y71H2_388.d^7^, *yars-1*^7^,**ZK809.3^7^
** No Epistasis**
* ant-1.1*^7^,* asg-2*^4^,**C18E9.4/.10^4^, C26B2.2^4^, C27B7.7^4^, C32H11.5^4^, C33A12.1^7^, C33F10.12^4^, C35A11.3^4^, C47E12.2^7^, *cec-3*^4^,* clec-186*^4^,* clec-227*^4^,* cyc-2.1*^7^,* cyp-33E2*^7^,**D1054.8^4^, E03H12.5^4^, *egl-3*^4^,**F29C4.2^7^, F35D2.3^4^, F40F8.5^4^, F43G9.1^4^, F49F1.12^4^, F55B11.5/.3^4^, *gcy-29*^4^,**H06H21.8^4^, *idh-1*^4^,**K07H8.1^4^, K10B4.3^4^, *max-1*^4^,* mecr-1*^4^,* mrpl-12*^7^,* mrps-33*^7^,* mrps-9*^7^,* nas-38*^4^,* nduf-2.2*^7^,* nhr-14*^4^,* nhr-154*^4^,* pfn-2*^4^,* pghm-1*^4^,* pup-2*^4^,* rnf-5*^4^,* rpl-30*^6^,* rpl-6*^6^,* rpl-9*^6^,* rps-10*^6^,* rps-26*^6^,* rps-6*^6^,* set-15*^4^,* set-18*^8^,* sid-2*^4^,* sru-17*^4^,* srw-20*^4^,**T05A1.4^4^, T26H5.1^4^, *tag-174*^7^,* tag-60*^4^,* tba-7*^7^,* ubh-4*^4^,* ucr-1*^7^,* unc-83*^4^,**Y119D3_463.b^7^, Y37D8A.12^4^, Y37D8A18^7^, Y39A3C_82.a^7^, Y43F4B.7^7^, Y43F8B.12^4^, Y46H3C.6^4^, Y53F4B.23^4^, Y53G8A_1734.g^7^, Y53G8A_2702.a^7^, Y53G8A_9248.b^7^, Y53G8A_9248.c^7^, Y53G8A_9248.d^7^, Y54E5A.2^8^, Y55F3B_743.b^7^, Y71H2_378.a^7^, Y71H2_385.b^7^, Y71H2_388.d^7^, Y92C3A.1^4^, *yars-1*^7^,**ZK809.3^7^

**Table 2.** Comparison of Methods Used in Genome-Wide RNAi Longevity Screens. A Summary of the Methods Used in the First 4 Papers Describing Genome-Wide RNAi Longevity Screens. The *fer-15(b26); fem-1(hc17)* Strain is Temperature-Sensitive Sterile Strain Allowing for Screening to be Performed in the Absence of FUdR. The *rrf-3(pk426)* Strain is Sensitive to RNAi. Some Studies were Performed in one Strain for the Initial Screen (Initial) and Hits were Validated in a Secondary Strain. The Stage at which RNAi was Initiated, Temperature, Presence of Absence of FUdR, Chromosomes Examined (CH) and Mutants Used for Longevity Epistasis Tests are Shown. ^a^ Lifespan Experiments were Performed at 20°C for the Screen, But Additional Lifespan EXPERIMENTS were Done 25°C. ^b^ *fer-15; fem-1* Worms were Developed at 25°C and Kept at Either 20 or 25°C for the Remainder of the Screen. Follow-up Experiments were Done at 20°C. ^c^ Lifespan Experiments were Performed at 25°C for the Screen, But Validation in *rrf-3* allowed Gravid Worms on RNAi Bacteria to Lay Eggs at 15°C for 12 Hrs, Development of Progeny at 25°C, and Subsequent Propagation at 20°C or 25°C

Paper	Initial/Secondary Strain	RNAi	Temp. (°C)	FUdR	CH	Epistasis
Dillin	N2/N2	Egg	25	NA	I	*daf-16(mu86)* *daf-2(e1370)*
Lee	N2/N2	L1	20^a^	+	I/II	*daf-16(mgDf47)*
Hansen	*fer-15; fem-1 /N2*	Egg	20 and 25^b^	-	ALL	*daf-16(mu86)* *daf-2(e1370)* *glp-1(e2141)* *daf-12(rh61rh411)* *eat-2(ad1116)*
Hamilton	N2/*rrf-3*	L1	25^c^	+	ALL	*daf-16(mgDf47)* *sir-2.1(ok434)*
